# A unique cytosolic activity related but distinct from NQO1 catalyses metabolic activation of mitomycin C

**DOI:** 10.1054/bjoc.1999.1096

**Published:** 2000-03-06

**Authors:** P Joseph, A K Jaiswal

**Affiliations:** Department of Pharmacology, Baylor College of Medicine, One Baylor Plaza, Houston, TX, 77030, USA

**Keywords:** mitomycin C, a unique cytosolic activity, NAD(P)H:quinone oxidoreductase1 (NQO1), mechanism of activation, DNA cross-linking

## Abstract

Mitomycin C (MMC) is a prototype bioreductive drug employed to treat a variety of cancers including head and neck cancer. Among the various enzymes, dicoumarol inhibitable cytosolic NAD(P)H:quinone oxidoreductase1 (NQO1) was shown to catalyse bioreductive activation of MMC leading to cross-linking of the DNA and cytotoxicity. However, the role of NQO1 in metabolic activation of MMC has been disputed. In this report, we present cellular and animal models to demonstrate that NQO1 may play only a minor role in metabolic activation of MMC. We further demonstrate that bioreductive activation of MMC is catalysed by a unique cytosolic activity which is related but distinct from NQO1. Chinese hamster ovary (CHO) cells were developed that permanently express higher levels of cDNA-derived NQO1. These cells showed significantly increased protection against menadione toxicity. However, they failed to demonstrate higher cytotoxicity due to exposure to MMC under oxygen (normal air) or hypoxia, as compared to the wild-type control CHO cells. Disruption of the NQO1 gene by homologous recombination generated NQO1–/– mice that do not express the NQO1 gene resulting in the loss of NQO1 protein and activity. The cytosolic fractions from liver and colon tissues of NQO1–/– mice showed similar amounts of DNA cross-linking upon exposure to MMC, as observed in NQO1+/+ mice. The unique cytosolic activity that activated MMC in cytosolic fractions of liver and colon tissues of NQO1–/– mice was designated as cytosolic MMC reductase. This activity, like NQO1, was inhibited by dicoumarol and immunologically related to NQO1. © 2000 Cancer Research Campaign


*British Journal of Cancer* (2000) **82**(7), 1305–1311
© 2000 Cancer Research Campaign
DOI: 10.1054/ bjoc.1999.1096, available online at http://www.idealibrary.com on IDEAL®

### A unique cytosolic activity related but distinct from NQO1 catalyses metabolic activation of mitomycin C

**P Joseph and AK Jaiswal**

Department of Pharmacology, Baylor College of Medicine, One Baylor Plaza, Houston, TX 77030, USA

**Summary** Mitomycin C (MMC) is a prototype bioreductive drug employed to treat a variety of cancers including head and neck cancer. Among the various enzymes, dicoumarol inhibitable cytosolic NAD(P)H:quinone oxidoreductase1 (NQO1) was shown to catalyse bioreductive activation of MMC leading to cross-linking of the DNA and cytotoxicity. However, the role of NQO1 in metabolic activation of MMC has been disputed. In this report, we present cellular and animal models to demonstrate that NQO1 may play only a minor role in metabolic activation of MMC. We further demonstrate that bioreductive activation of MMC is catalysed by a unique cytosolic activity which is related but distinct from NQO1. Chinese hamster ovary (CHO) cells were developed that permanently express higher levels of cDNA-derived NQO1. These cells showed significantly increased protection against menadione toxicity. However, they failed to demonstrate higher cytotoxicity due to exposure to MMC under oxygen (normal air) or hypoxia, as compared to the wild-type control CHO cells. Disruption of the NQO1 gene by homologous recombination generated NQO1–/– mice that do not express the NQO1 gene resulting in the loss of NQO1 protein and activity. The cytosolic fractions from liver and colon tissues of NQO1–/– mice showed similar amounts of DNA cross-linking upon exposure to MMC, as observed in NQO1+/+ mice. The unique cytosolic activity that activated MMC in cytosolic fractions of liver and colon tissues of NQO1–/– mice was designated as cytosolic MMC reductase. This activity, like NQO1, was inhibited by dicoumarol and immunologically related to NQO1. © 2000 Cancer Research Campaign

**Keywords**: mitomycin C; a unique cytosolic activity; NAD(P)H:quinone oxidoreductase1 (NQO1); mechanism of activation; DNA cross-linking

Bioreductive chemotherapy plays an important role in treatment of many human diseases including certain types of cancer (Sartorelli, 1988; Ross et al, 1994; Workman, 1994; Rauth et al, 1997). The development of these drugs is based upon specific reductase enzymes and the identification of tumour types that are rich in those enzymes. In addition, it is also based on differences in oxygen content and cellular pH between normal and tumour tissues (Ross et al, 1994). Several quinones (e.g. mitomycin C (MMC) and indoloquinone EO9) and dinitrophenylaziridine CB1954 occupy a special place in bioreductive therapy because of their tendency to undergo reductive activation by different reductases in aerobic and hypoxic conditions. MMC and its analogues are effective against several tumour tissue types including colon, breast and head and neck (Verweij and Pinedo, 1990).

Among the various reductases, the role of NAD(P)H:quinone oxidoreductase1 (NQO1) in bioreductive chemotherapy was extensively studied. Most of the studies were done for MMC. However, NQO1 enzyme has also been demonstrated for the activation of CB1954 (Knox et al, 1988), diaziquinone (Siegel et al, 1990), and the indoloquinone EO9 (Workman, 1994). Bioreductive metabolism leading to the formation of electrophilic metabolites capable of binding with DNA to cause its damage is the basic cause of cytotoxicity and anti-tumour activity of MMC (Iyer and Szybalski, 1964). Several studies used dicoumarol inhibition of NQO1 to demonstrate that NQO1 is important for the cytotoxicity of MMC under aerobic conditions (Siegel et al, 1990; Dulhanty and Whitmore, 1991; Marshall et al, 1991). However, the actual involvement of NQO1 enzyme in the activation of MMC remains controversial (Workman, 1994). Although as mentioned earlier, there are a number of model systems where aerobic resistance to MMC correlates with a loss of NQO1 activity (Fitzsimmons et al, 1996), a lack of correlation was reported in the ‘Oxford’ panel of 15 cell lines (Robertson et al, 1992). More recent data using cells expressing higher levels of cDNA-derived NQO1 failed to demonstrate increased sensitivity to MMC, as compared to cells of similar origin containing normal levels of NQO1 (Powis et al, 1995; Joseph et al, 1996). The role of NQO1 in activation of MMC under hypoxic conditions is also questionable (Workman, 1994). This is clearly evident by the observation of high sensitivity of human colon carcinoma (BE) cells that lack NQO1 to MMC and indoloquinone EO9 in hypoxia (Plumb and Workman, 1994).

In this report, we present evidence to demonstrate that a unique cytosolic enzyme distinct from NQO1 catalyses reductive activation of MMC. These include: (1) the CHO cells permanently overexpressing cDNA-derived NQO1 failed to demonstrate higher sensitivity to MMC, as compared to control CHO cells; and (2) the cytosolic extracts from liver and colon of NQO1–/– mice showed more or less similar amounts of MMC-induced DNA cross-linking, as compared with cytosolic extracts of wild-type (NQO1+/+) mice. Further experiments revealed that cytosolic MMC reductase is inhibited by dicoumarol and is immunologically related to cytosolic NQO1.

*Received 30 June 1999*
*Revised 5 November 1999*
*Accepted 8 November 1999*

*Correspondence to:* AK Jaiswal

### MATERIALS AND METHODS

### Materials

The Chinese hamster ovary (CHO) cells were obtained from American Type Culture Collection (ATCC, Rockville, MD, USA). MMC was a gift from Bristol Myers Squibb (Princeton, NJ, USA). Polyclonal antibodies against purified full-length NQO1 protein was raised in rabbits in our laboratory. The NQO1 antibodies cross-react with cytosolic NQO1 and NQO2 proteins from rodents and human (Jaiswal et al, 1990; Shaw et al, 1991).

### Development of CHO cells stably expressing high levels of cytosolic NQO1 activity

The human NQO1 cDNA was subcloned in pED4 vector (Kaufman et al, 1991) to generate pED4-NQO1 recombinant plasmid. The CHO cells were grown in 5% carbon dioxide at 37°C in Iscove's Dulbecco's modified essential medium (DMEM) containing 10% fetal bovine serum, 0.1 mM hypoxanthine, 0.01 mM thymidine, 50 μl $ml^{-1}$ streptomycin and 2 mM glutathione. pED4-NQO1 plasmid DNA was linearized by digestion with Nde1 and transfected in CHO cells as described previously (Shaw et al, 1991). Sixty-two hours after transfection, the cells were washed with 1 × phosphate-buffered saline (PBS) trypsinized and plated in medium deficient in thymidine and hypoxanthine at different dilutions of 1:5, 1:10, 1:20 and 1:40. A portion of cells were analysed for NQO1 activity by a previously described method (Shaw et al, 1991). The cells were allowed to grow in medium deficient in thymidine and hypoxanthine for 14 days. During this period, the medium was changed every third day. After 14 days, the colonies were visible. They were picked and expanded in 24-well plates. Selection of CHO cells continued in medium containing methotrexate at concentrations of 0.02, 0.1 and 0.5 μM. All the clones selected at various stages of methotrexate treatment were analysed for NQO1 activity. Three of these clones CHO (NQO1-13), CHO (NQO1-38) and CHO (NQO1-3682) expressing 13-, 38- and 3682-fold higher levels of NQO1, as compared with wild-type control CHO cells, were selected for further studies. The selected CHO cell clones were grown in medium deficient in methotrexate for 4 weeks without loss of NQO1 activity.

### Cytotoxicity assays

The wild-type control CHO cells and the three CHO cell clones expressing varying levels of cDNA-derived NQO1 were grown in dishes without methotrexate. The various cells were treated with different concentrations of MMC and mendione (vitamin K). The growth/survival of cells following 3 days of exposure to drugs was measured by procedures as described (Skehan et al, 1990; Zilfou et al, 1995). In addition, the cytotoxicity of drugs was also determined by colony formation assay as described previously (Sakai et al, 1995). Briefly, the CHO cells expressing different levels of NQO1 were grown in monolayer, trypsinized and plated at a density of 200 cells per 60-mm Petri dish in medium containing the various concentrations of MMC. After 24 h, the medium containing MMC was replaced with fresh medium without MMC. The hypoxic treatment of MMC was done for 1 h. The cells were allowed to grow for 12–15 days into colonies. The colonies were stained and counted. The drug concentration to produce 50% reduction in colony formation was determined.

### Generation of NQO1–/– mice

We used gene targeting method to disrupt the NQO1 gene and generated NQO1–/– mice that do not express NQO1 protein and activity (Radjendirane et al, 1998). The development of NQO1–/– mice was normal. The NQO1+/+ and NQO1–/– mice were bred to generate sufficient mice for MMC sensitivity assays. The levels of dicoumarol-sensitive cytosolic NQO1 activity in the various tissues of wild-type (NQO1+/+) and NQO1–/– mice were measured. The NQO1 activity (nmol of 2,6-dichlorophenolindophenol reduced $min^{-1}$ $mg^{-1}$ cytosolic protein) dropped from 515.3 ± 37.2 in colon of wild-type mice to zero levels in NQO1–/– mice. The NQO1 activity also dropped significantly (> 85%; NQO1+/+, 136.5 ± 10.2; NQO1–/–, 19.6 ± 4.1) in liver. The residual amounts of NQO1 activity observed in livers of NQO1-null mice is due to NQO1 related protein(s) rather than NQO1 (Radjendirane et al, 1998). This is because NQO1 protein was absent in livers of NQO1–/– mice as determined by Western analysis (Radjendirane et al, 1998).

### Metabolic activation of MMC in cytosolic fractions from wild-type (NQO1+/+) and NQO1–/– mice as determined by DNA cross-linking

The cytosolic fractions from liver and colon tissues of wild-type (NQO1+/+) and NQO1–/– mice were prepared by standard procedures. These cytosolic fractions were absolutely free of microsomal contamination as no activity of microsomal-specific enzyme NADPH: cytochrome P450 reductase was detected in the various cytosolic fractions (data not shown). The cytosolic fractions from wild-type (NQO1+/+) mice containing one unit of NQO1 activity (equivalent to one μmol of 2,6-dichlorophenoindophenol reduced in 1 min) and similar amounts of cytosolic proteins from NQO1–/– mice were incubated with 23 base pair of double-stranded oligonucleotides containing MMC binding site and DNA cross-linking assays performed by procedure as described (Joseph et al, 1996). The nucleotide sequence of the 23 base pair oligonucleotide was 5′-CTACATCGTGTCATGCACAGGAT-3′. The complementary strands were mixed in equal amounts and annealed by heating to 70°C and slowly cooling to room temperature. The 3′-end of the top strand was selectively labelled by Klenow enzyme in the presence of [$\alpha$-$^{32}$P]dCTP. The labelled duplexes were purified on a 15% polyacrylamide gel and used for incubation with the cytosolic extracts for cross-linking by activated MMC. The incubation mixture included 100 mM phosphate buffer (pH 5.8), cytosolic protein, 1 mM NADH, 100 μM MMC, 5 μM FAD, 0.01% Tween-20 and 0.18 mg bovine serum

**Table 1** Mitomycin C-induced cytotoxicity of CHO and CHO-NQO1 cells as determined by colony formation assay

| S. No. | Cells | NQO1 activity | Mitomycin C $IC_{50}$ |
|---|---|---|---|
| 1 | CHO (W) | 0.02 ± 0.01 | 75 ± 4 |
| 2 | CHO-NQ01-(13) | 0.27 ± 0.03 | 71 ± 5 |
| 3 | CHO-NQ01-(38) | 11.54 ± 0.10 | 73 ± 4 |
| 4 | CHO-NQO1-(3682) | 73.65 ± 3.10 | 59 ± 2 |

NQO1 activity, μmol of 2,6-dichlorophenolindophenol reduced $min^{-1}$ $mg^{-1}$ protein. Mitomycin C (MMC) $IC_{50}$ μmol of MMC required to kill 50% of cells in cytotoxicity assays as determined by the procedures as described (Sakai et al, 1995).

 © 2000 Cancer Research Campaign albumin. Reactions were terminated by the addition of ethanol, and the precipitated oligomer duplexes were denatured by heating at 95°C for 5 min in DNA sequencing dye containing formamide. The samples were analysed on a 15% denaturing polyacrylamide gel, and cross-linked and unmodified oligomers were detected by autoradiography.

### Further characterization of the unique cytosolic activity

We used polyclonal antibodies against full-length NQO1 protein in DNA cross-linking assays to determine if the unique cytosolic activity that activates MMC leading to DNA cross-linking is related to NQO1. The cytosolic extracts from human colon carcinoma (HT29) cells containing one unit of NQO1 activity (reduction of one μmol of 2,6-dichlorophenolindophenol in 1 min) were incubated with either pre-immune serum or antibodies against NQO1 in various concentrations for antigen/antibody reactions. Half of the incubation mixture was analysed for NQO1 activity (2,6-dichlorophenolindophenol reduction) and another half analysed for its capacity to catalyse MMC induced DNA cross-linking. Similar experiments were also performed with colon cytosolic extracts of NQO1+/+ and NQO1–/– mice.

### RESULTS

The transfection of CHO cells with pED4-NQO1 plasmid resulted in generation of a series of CHO cell clones that permanently express higher levels of NQO1 (Table 1). Three of these clones

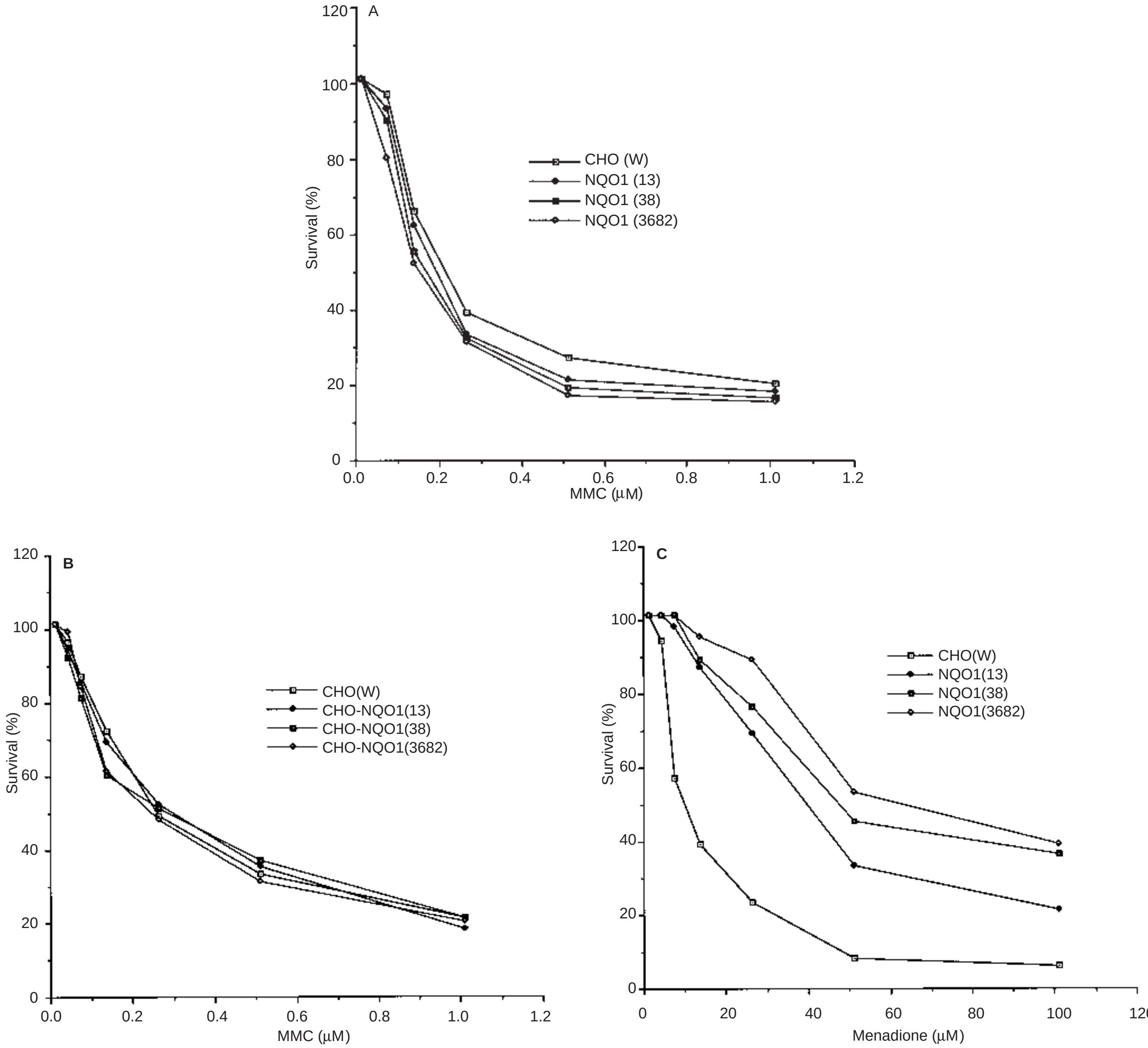

**Figure 1** Sensitivity of CHO cells expressing different levels of cDNA-derived NQO1 to MMC and menadione. The wild-type CHO (W) cells and CHO cells permanently expressing higher levels of cDNA-derived NQO1 were grown in dishes and treated with MMC or menadione. The growth/survival of cells following three days of exposure to drugs was measured. (**A**) Treatment with MMC; (**B**) treatment with MMC in hypoxia; (**C**) treatment with menadione. All values are mean of three independent experiments

*© 2000 Cancer Research Campaign*

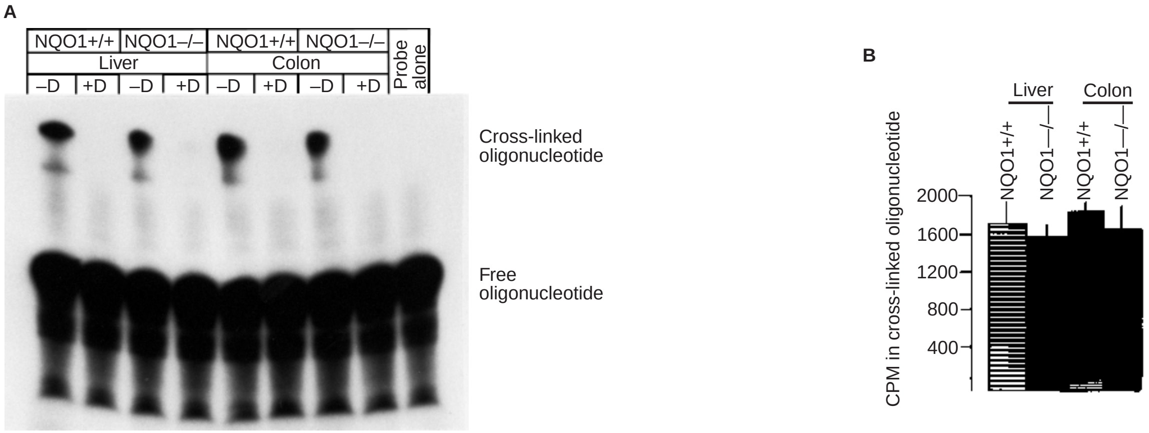

**Figure 2** Mitomycin C-induced inter-strand DNA cross-linking. (**A**) Cytosolic extracts from wild-type (NQO1+/+) and NQO1–/– mice were incubated with $^{32}$P-labelled 23 base pairs of oligonucleotides containing the mitomycin C binding site and 100 $\mu$M mitomycin C in absence and presence of dicoumarol by procedures as described in Materials and Methods. The reaction was stopped with DNA sequencing dye containing formamide, heated to denature the DNA and separated on 15% denaturing polyacrylamide gel. The gel was dried and autoradiographed. –D, No dicoumarol; +D, 10 $\mu$M dicoumarol. (**B**) The spots containing cross-linked oligonucleotides were cut out and counted. The results are presented as mean ± s.e.m. of CPM from three independent experiments

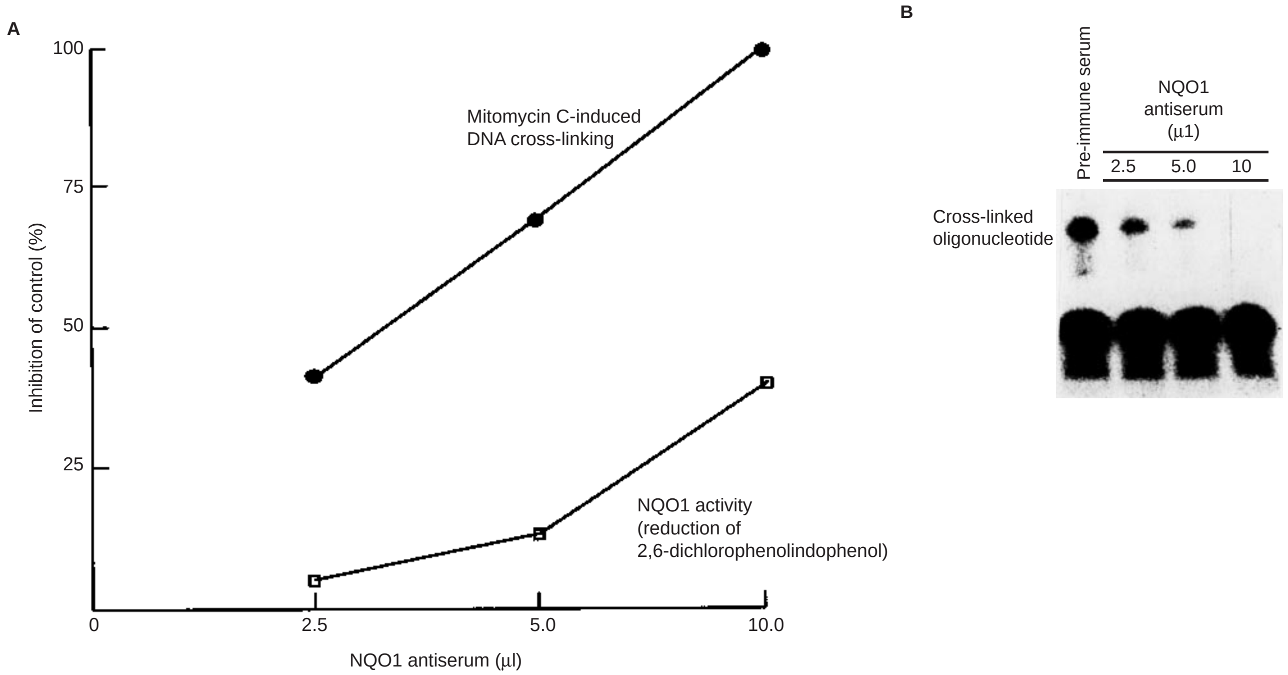

**Figure 3** (**A**) Comparison of per cent inhibition of mitomycin C induced DNA cross-linking and cytosolic NQO1 activity from human colon carcinoma HT29 cells under similar conditions. The cytosolic extracts from HT29 cells containing one unit of NQO1 activity (2,6-dichlorophenolindophenol) were treated with either pre-immune serum (10 µl) or anti-NQO1 serum (in different concentrations) for 1 h at 4°C. Half of the incubation mixture was analysed for NQO1 (2,6-dichlophenolindophenol reduction) and another half analysed for its capacity to catalyse mitomycin C (MMC) induced DNA cross-linking. The cross-linked bands were cut out and counted for per cent inhibition. The inhibition of NQO1 activity and MMC induced DNA cross-linking by anti-NQO1 antibodies are represented as per cent of control results obtained with cytosolic extracts treated with pre-immune serum. The data are presented as mean of three independent experiments. (**B**) Inhibition of MMC-induced DNA cross-linking by polyclonal antibodies against purified human NQO1 (NQO1 antiserum). The cytosolic extract from HT29 cells containing one unit of NQO1 activity (2,6-dichlorophenolindophenol) was treated with either pre-immune serum (10 µl) or anti-NQO1 serum (in different concentrations) for 1 h at 4°C and analysed for their capacity to catalyse mitomycin C (MMC) induced DNA cross-linking expressing 13-, 38- and 3682-fold higher levels of NQO1 were used for MMC cytotoxicity assays. Two methods were used to assess the cytotoxicity of the various CHO cell clones to MMC. These included: (1) inhibition of growth of CHO cells as determined by SRB colourimetric assay, and (2) colony formation. The results are shown in Figure 1 and Table 1. The CHO cells expressing higher levels of NQO1 showed more or less similar survival rate as wild-type control upon treatment with different concentrations of MMC (Figure 1A). Similar results were observed upon treatment of CHO cells with MMC in hypoxia (Figure 1B). However, replacement of MMC with menadione showed significant protection of CHO cells expressing higher levels of NQO1, as compared with wild-type control cells (Figure 1C). The results also indicated absence of a positive correlation

 *© 2000 Cancer Research Campaign*

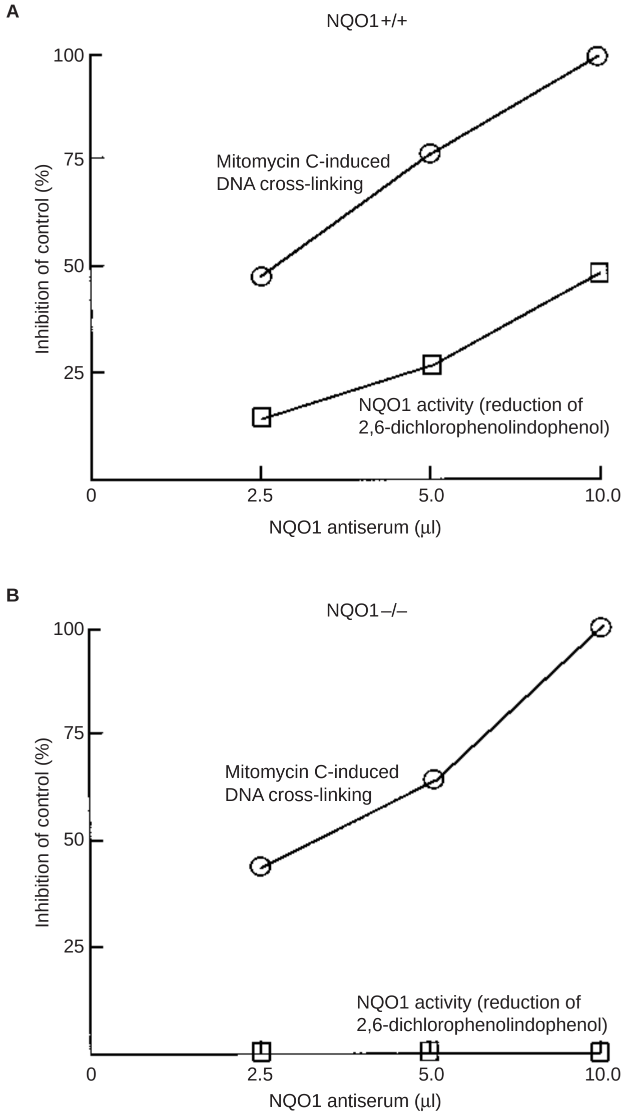

**Figure 4** Inhibition of mitomycin C-induced DNA cross-linking and cytosolic NQO1 activity from wild-type (NQO1+/+) and knock out (NQO1–/–) mice colon. The cytosolic extracts from colon of wild-type and NQO1–/– mice were treated with either pre-immune serum (10 µl) or with the various concentrations of anti-NQO1 serum for 1 h at 4°C and analysed for mitomycin C (MMC) induced DNA cross-linking. The data are mean ± of three independent experiments between NQO1 levels in CHO cells and $IC_{50}$ of MMC as determined by colony formation assays (Table 1). The 3682-fold increase in NQO1 activity in CHO cells resulted only in less than twofold increase in sensitivity of these cells to MMC (Table 1).

MMC-induced inter-strand DNA cross-linking was observed with cytosolic extracts from both wild-type (NQO1+/+) and NQO1–/– mice tissues (Figure 2 A and B). However, the amount of DNA cross-linking was slightly lower in case of NQO1–/– mice tissues as compared to NQO1+/+ mice. This reduction in DNA cross-linking was no more than 15% of wild-type. The MMC-induced DNA cross-linking in wild-type as well as NQO1–/– tissues was inhibited by dicoumarol (Figure 2).

The results of antibody inhibition are shown in Figures 3 and 4. The antibodies inhibited both NQO1 activity and MMC-induced DNA cross-linking in human colon carcinoma (HT29) cells and wild-type (NQO1+/+) and knock out (NQO1–/–) mice. However, the inhibition kinetics of NQO1 activity was significantly different than that of MMC reductase-induced DNA cross-linking. The 2.5 µl of NQO1 antibodies inhibited 5.5% of NQO1 activity in HT29 cells, as compared to 43% inhibition of MMC-induced DNA cross-linking (Figure 3). Similarly, 10 µl of NQO1 antibodies inhibited 100% of MMC-induced DNA cross-linking, as compared to only 41% inhibition of the NQO1 activity in HT29 cells (Figure 3). Similar results were also observed with cytosolic extracts from colon of NQO1+/+ mice (Figure 4A). Therefore, the NQO1 antiserum inhibited MMC-induced DNA cross-linking more efficiently than NQO1 activity in both HT29 cells and NQO1+/+ mice. The antibodies against NQO1 also inhibited MMC induced DNA cross-linking in NQO1 deficient (NQO1–/–) mice colon (Figure 4B).

### DISCUSSION

Bioreductive chemotherapy may be the most successful treatment for certain types of cancer (Sartorelli, 1988; Ross et al, 1994; Workman, 1994; Rauth et al, 1997). This is because it is based on differences between normal and tumour tissues in enzyme levels, oxygen content and pH. The enzymes which are expressed at higher levels in tumours may activate the drugs for killing the tumour cells. However, similar activation in normal cells may be at lower levels which can be detoxified. Therefore, effective dosage of drugs could be administered with drastic damage to tumour cells and less or no toxicity to normal cells. In addition, the existence of hypoxic regions is a major problem in radiation therapy, as oxygen is a radiation sensitizer. Therefore, hypoxic parts of the tumours often survive radiation treatment, and may be centre for a recurrent tumour. Bioreductive agents such as MMC may be more active in these hypoxic regions of the tumours (Kennedy et al, 1980; Stratford and Stephens, 1989). The administration of such bioreductive agents as adjuvant to radiation may be favourable for killing solid tumour cells. Identification of enzyme(s) that metabolically activate anti-tumour drugs like MMC is one step forward in this direction.

In this report, we studied the role of NQO1 and other cytosolic enzyme(s) in metabolic activation of MMC. The results revealed that cytosolic NQO1 plays an insignificant role in metabolic activation of MMC. The CHO cells stably expressing higher levels of cDNA-derived NQO1 did not demonstrate increased cytotoxicity upon exposure to MMC. This is in agreement with the earlier report by Gustafson et al (1996). In addition, the loss of NQO1 due to disruption of NQO1 gene did not lead to a significant difference in sensitivity of NQO1–/– mice to MMC, as compared with wild-type (NQO1+/+) mice. Both wild-type (NQO1+/+) and NQO1–/– mice showed more or less similar response to MMC exposure. Furthermore, the cytosolic fractions from liver and colon of NQO1–/– mice deficient in NQO1 protein/activity catalysed high affinity reductive activation of MMC which led to inter-strand DNA cross-linking. The small amount of differences between wild-type (NQO1+/+) and NQO1–/– mice may be due to experimental variations or due to very low affinity of NQO1 for MMC.

The results on MMC-induced DNA cross-linking also revealed that cytosolic fractions of wild-type (NQO1+/+) and NQO1–/– mice contain an activity that catalysed metabolic activation of MMC which led to DNA cross-linking. This activity is designated as cytosolic MMC reductase. The cytosolic MMC reductase activity was inhibited by low concentrations of dicoumarol, an inhibitor of NQO1. This indicated that cytosolic MMC reductase activity may be related to NQO1. Further experiments using polyclonal antibodies against full-length NQO1 protein confirmed this. The NQO1 antibodies inhibited both NQO1 activity catalysed reduction of 2,6-dichlorophenolindophenol and cytosolic MMC reductase activity catalysed MMC-induced DNA cross-linking. However, NQO1 antibodies were more effective in inhibiting the MMC-induced DNA cross-linking, as compared to reduction of 2,6-dichlorophenolindophenol. This may be due to lower concentration of MMC reductase activity as compared to the NQO1 activity. It is for this reason that 10 μl of NQO1 antiserum inhibited 100% of MMC-induced DNA cross-linking, as compared to 41% inhibition of NQO1 activity.

The MMC reductase activity is expected to be a new member of NQO gene family. Four gene loci encode for the various members of the NQO gene family (Jaiswal, 1988). Two of these loci (NQO1 and NQO2) have been cloned and sequenced (Jaiswal et al, 1988, 1990). NQO2 is a second member of the NQO gene family that requires NRH instead of NAD(P)H as a cofactor (Wu et al, 1997). MMC reductase activity may not be due to NQO2 because it requires NAD(P)H and not NRH as cofactor (Joseph et al, 1996).

Much of the literature suggests that cytosolic NQO1 and NQO2 protect cells against quinone-induced redox cycling and oxidative stress (Riley and Workman, 1992; Talalay et al, 1995). This is supported by observations in the present report that cytosolic NQO1 protected CHO cells against menadione while remaining more or less insensitive to MMC cytotoxicity. Based on the present results, we hypothesize that NQO1 may play no, or an insignificant, role in the activation of MMC but may exclusively protect cells against redox cycling and oxidative stress. NQO1–/– mice represent a valuable tool to confirm this hypothesis using other drugs such as CB1954 and EO9. We further hypothesize that MMC reductase catalyses metabolic activation of MMC and other drugs and xenobiotics containing quinone structures.

In summary, we used cellular and NQO1–/– mice models to demonstrate that cytosolic NQO1 may not activate MMC. We have characterized a unique cytosolic (MMC reductase) activity that catalyses metabolic activation of MMC which led to DNA cross-linking. This activity is distinct from NQO1 but related to NQO1. Purification and cloning of MMC reductase activity should reveal further information on this protein and its role in bioreductive drugs activation.

### ACKNOWLEDGEMENTS

We are thankful to our colleagues for suggestions and discussions. This work was supported by NIH grants RO1 ES07943 and RO1 CA81057.

### REFERENCES

Dulhanty AM and Whitmore GF (1991) Chinese hamster ovary cells resistant to mitomycin C under aerobic but not hypoxic conditions are deficient in DT-diaphorase. *Cancer Res* **51**: 1860–1865

Fitzsimmons SA, Workman P, Grever M, Paull K, Camalier R and Lewis AD (1996) Reductase enzyme expression across the National Cancer Institute Tumor Cell Line panel: correlation with sensitivity to mitomycin C and EO9. *J Natl Cancer Inst* **21**: 1161–1163

Gustafson DL, Beall HD, Bolton EM, Ross D and Waldren CA. Expression of human NAD(P)H:quinone oxidoreductase (DT-diaphorase) in Chinse hamster ovary cells: effect on the toxicity of antitumor quinones. *Mol Pharmacol* **50**: 728–735

Iyer VN and Szybalski W (1964) Mitomycins and porfiromycin: chemical mechanism of activation and cross-linking of DNA. *Science* **145**: 55–58

Jaiswal AK, McBride OW, Adensik M and Nebert DW (1988) Human dioxin-inducible cytosolic NAD(P)H:quinone oxidoreductase. *J Biol Chem* **263**: 13572–13578

Jaiswal AK, Burnett P, Adesnik M and McBride OW (1990) Nucleotide and deduced amino acid sequence of a human cDNA (NQO2) corresponding to a second member of the NAD(P)H:quinone oxidoreductase gene family. Extensive polymorphism at the NQO2 gene locus on chromosome 6. *Biochemistry* **29**: 1899–1906

Joseph P, Xu Y and Jaiswal AK (1996) Non-enzymatic and enzymatic activation of mitomycin C. *Int J Cancer* **65**: 263–271

Kaufman RJ, Davies MV, Wasley LC and Michnick D (1991) Improved vectors for stable expression of foreign genes in mammalian cells by use of the untranslated leader sequence from EMC virus. *Nucleic Acids Res* **19**: 4485–4490

Kennedy KA, Teicher BA, Rockwell S and Sartorelli AC (1980) The hypoxic tumor cell: a target for selective cancer chemotherapy. *Biochem Pharmacol* **29**: 1–8

Knox RJ, Boland MP, Friedlos F, Coles B, Southan C and Roberts JJ (1988) The nitroreductase enzyme in Walker cells that activates 5-(aziridin-1-yl)-2,4-dinitrobenzamide (CB 1954) to 5-(aziridin-1-yl)-4-hydroxyl-amino-2-nitrobenzamide is a form of NAD(P)H dehydrogenase (quinone) (EC 1.6.99.2). *Biochem Pharmacol* **37**: 4671–4677

Marshall RS, Paterson MC and Rauth AM (1991) DT-diaphorase and mitomycin C sensitivity in nontransformed cell strains derived from members of a cancer prone family. *Carcinogenesis* **12**: 1175–1180

Plumb JA and Workman P (1994) Unusually marked hypoxic sensitization to indoloquinone EO9 and mitomycin C in a human colon-tumour cell line that lacks DT diaphorase activity. *Int J Cancer* **56**: 134–139

Powis G, Gasdaska PY, Gallegos A, Sherrill K and Goodman D (1995) Over-expression of DT-diaphorase in transfected NIH 3T3 cells does not lead to increased anticancer quinone drug sensitivity: a questionable role for the enzyme as a target for bioreductively activated anticancer drugs. *Anticancer Res* **15**: 1141–1146

Radjendirane V, Joseph P, Lee H, Kimura S, Klein-Szanto AJP, Gonzalez FJ and Jaiswal AK (1998) Disruption of the DT diaphorase (NQO1) gene in mice leads to increased menadione toxicity. *J Biol Chem* **273**: 7382–7389

Rauth AM, Goldberg Z and Misra V (1997) DT-diaphorase: possible roles in cancer chemotherapy and carcinogenesis. *Oncol Res* **9**: 339–349

Riley RJ and Workman P (1992) DT-diaphorase and cancer chemotherapy. *Biochem Pharm* **43**: 1657–1669

Robertson N, Stratford IJ, Houlbrook S, Carmichael J and Adams GE (1992) The sensitivity of human tumor cells to quinone bioreductive drugs: what role for DT-diaphorase? *Biochem Pharmacol* **44**: 409–412

Ross D, Beall H, Traver RD, Siegel D, Phillips RM and Gibson NW (1994) Bioactivation of quinones by DT-diaphorase, molecular, biochemical, and chemical studies. *Oncol Res* **6**: 493–500

Sakai A, Miyata N and Takahashi A (1995) Initiating activity of quinones in the two-stage transformation of Balb/3T3 cells. *Carcinogenesis* **16**: 477–481

Sartorelli AC (1988) Therapeutic attack of solid tumors. *Cancer Res* **48**:775–778

Shaw PM, Reiss A, Adesnik M, Nebert DW, Schembri J and Jaiswal AK (1991) The human dioxin-inducible NAD(P)H:quinone oxidoreductase cDNA-encoded protein expressed in COS-1 cells in identical to diaphorase 4. *Eur J Biochem* **195**: 171–176

Siegel D, Gibson NW, Perusch PC and Ross D (1990) Metabolism of diaziquone by NAD(P)H:(quinone acceptor) oxidoreductase (DT-diaphorase): role in diaziquone-induced DNA damage and cytotoxicity in human colon carcinoma cells. *Cancer Res* **50**: 7293–7300

Skehan P, Storeng R, Scudiero D, Monks A, McMahon J, Vistica D, Warren JT, Bokesch H, Kenney S and Boyd MR (1990) New colorimetric cytotoxicity assay for anticancer-drug screening. *J Natl Cancer Inst* **82**: 1107–1118

Stratford IJ and Stephens MA (1989) The differential hypoxic cytotoxicity of bioreductive agents determined in vitro by the MTT assay. *Int J Radiation Oncol Biol Phys* **16**: 973–976

Talalay P, Fahey JW, Holtzclaw WD, Prestera T and Zhang Y (1995) Chemoprotection against cancer by phase 2 enzyme induction. *Toxicol Lett* **82–83**: 173–179

Verweij J and Pinedo HH (1990) In: *Cancer Chemotherapy and Biological Modifyers, Annual 11*, Pinedo HM, Cabner BA and Longo DL (eds), pp. 63–73. Elsevier Science: Amsterdam.

 © 2000 Cancer Research Campaign

Workman P (1994) Enzyme-directed bioreductive drug development revisited: a commentary on recent progress and future prospects with emphasis on quinone anticancer agents and quinone metabolizing enzymes, particularly DT-diaphorase. *Oncol Res* **6**: 461–475

Wu K, Knox R, Sun XZ, Joseph P, Jaiswal AK, Zhang D, Deng PSK and Chen S (1997) Catalytic properties of NAD(P)H:quinone oxidoreductase2 (NQO2), a dihydronicotinamide riboside dependent oxidoreductase. *Arch Biochem Biophys* **345**: 221–228

Zilfou JT and Smith CD (1995) Differential interactions of cytochalasins with P-glycoprotein. *Oncol Res* **7**: 435–443

© 2000 Cancer Research Campaign
